# Synthesis, solvatochromism and crystal structure of 5-Methoxy-5,6-diphenyl-4,5-dihydro-2*H −*1,2,4-triazine-3-thione

**DOI:** 10.1186/1752-153X-7-130

**Published:** 2013-07-28

**Authors:** Behrooz Rezaei, Mehrnoosh Fazlollahi

**Affiliations:** 1 Department of Chemistry, Lorestan University, Khorramabad, Iran

**Keywords:** Triazine, Thione, Hydrogen bond, Electronic absorption spectra, Solvatochromic, Crystal structure, X- ray

## Abstract

**Background:**

For the development of properties in molecular crystals, such as electrical conductivities, magnetic properties, or non-linear optical properties, not only the electronic properties of molecules themselves matter, but also the molecular arrangements in the crystals are very important. Therefore, the design of the crystal structures and the control of molecular arrangements have attracted much attention in recent years. Among various ligands, triazine moieties have been especially interesting because of their biological, pharmacological and medicinal properties.

**Results:**

Crystal structure of 5-Methoxy-5,6-diphenyl-4,5-dihydro-2H-1,2,4-triazine-3-thione is Monoclinic, which consists of space group P21/c with a = 9.699(1), b = 8.500(1), c = 18.044(2) Å, β = 101.290(7)° and Z = 4, R1 = 0.0371 and wR2 = 0.1008 with 2456 reflections (I > 2σ(I*)). Intermolecular H bonds between NH groups are acting as donors and S atoms as acceptors.* There are also shorted face-to-face as well as edge to face π-π stacking interactions between the parallel aromatic rings. The behavior of solvatochromic of the mentioned compound that involved interhydrogen bonding was investigated by studying its electronic absorption spectra in pure organic solvents of different characters.

**Conclusions:**

The crystal structure of C_16_H_15_N_3_OS, shows the expected face-to-face π-π stacking interactions between aromatic rings of the neighbor chains in this compound. The centroid–centroid distance between the aromatic rings is 3.325 Å. It was found that the monomer of the ligand 5-Methoxy-5,6-diphenyl-4,5-dihydro-2H-1,2,4-triazine-3-thione, further extends into 3D networks via hydrogen bonding and pi-pi stacking interactions. The solvatochromic behavior of the title compound was also investigated by studying its spectra in a selection of different organic solvents. While progressing from the non-polar solvent to the polar one, the main intense band at 310 nm, which is due to the π–π* transition, was red shifted by 13 nm. Thus, the title compound showed positive solvatochromic behavior.

## Introduction

**Figure 1:**
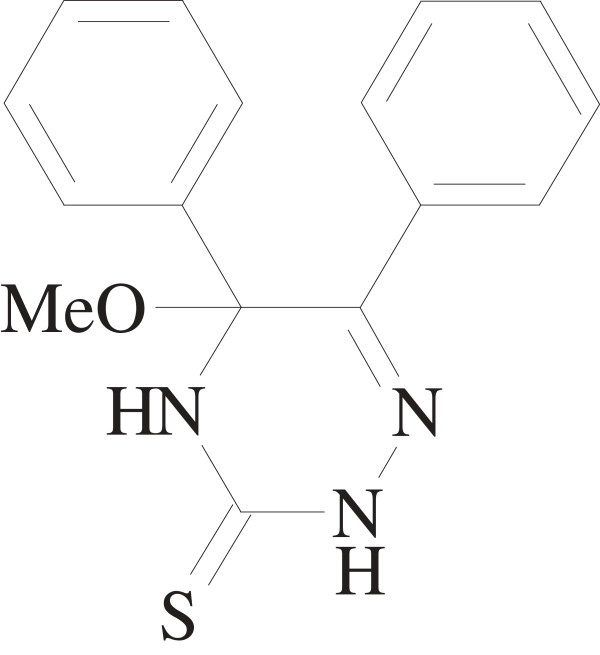
**Chemical structure of 5-Methoxy-5,6-diphenyl-4,5-dihydro-2*H*-1,2,4-triazine-3-thione.**

Numerous compounds containing the 1,2,4-triazine moieties are well known in natural materials and show interesting biological, pharmacological and medicinal properties. One of the important classes of N-containing heterocycles is the 3,5,6-trisubstituted-1,2,4-triazines. Some of them can be active as blood platelet aggregation inhibitors and others exhibit antiviral inhibitory activity (against influenza viruses for example), significant activity towards leukaemia and ovarian cancer, and anti-HIV activity [1-4]. Therefore, the design of molecule structures and control of molecular arrangements have attracted much attention in recent years [5]. In this paper, we wish to report the crystalline structure of 5-Methoxy-5,6-diphenyl-4,5-dihydro-2*H*-1,2,4-triazine-3-thione (Figure 1). In addition to structural, solvatochromic properties of the mentioned molecule are discussed.

## Results and discussion

**Figure 2:**
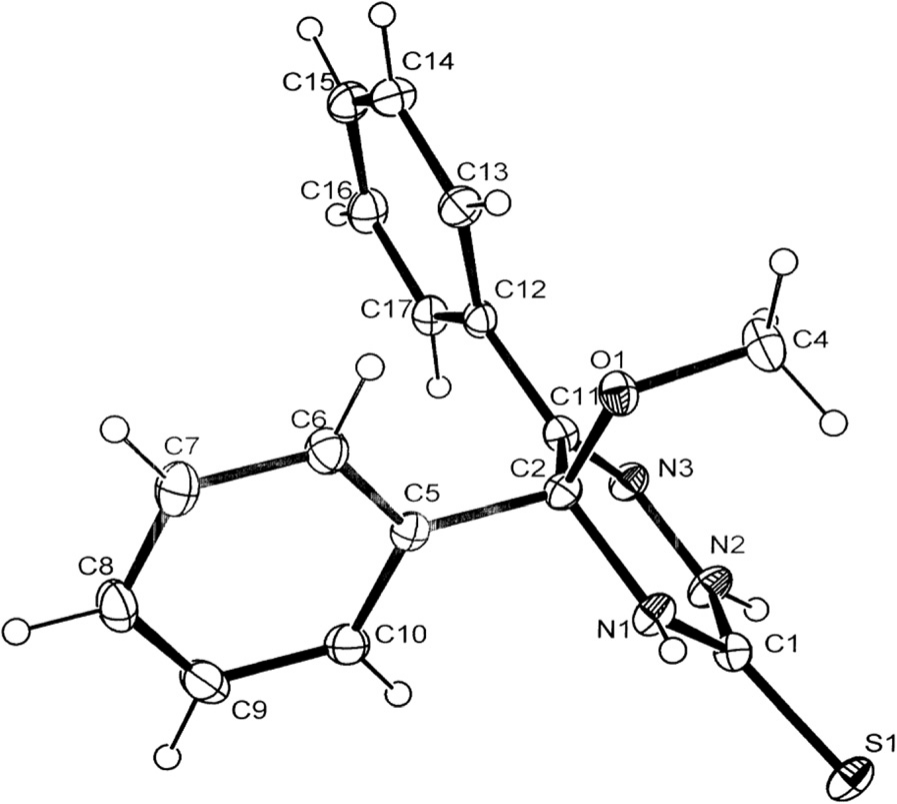
**The ORTEP diagram triazine-3-thione of 5-Methoxy-5,6-diphenyl-4,5-dihydro-2*H*-1,2,4- triazine-3-thione.**

The crystal structure 5-Methoxy-5,6-diphenyl-4,5-dihydro-2*H*-1,2,4-triazine-3-thione was solved by direct methods (SIR97) [6] and refined by Full-matrix least squares using the program SHELXTL-97 [7]. The H atoms were refined isotropically. The data had been collected by using a STOE IPDSII. The molecular structure of the title compound is presented in Figure 2. The corresponding crystal and structure refinement data are summarized in Table 1 and all atomic coordinates and equivalent isotropic displacement parameters are given in Table 2. The compound crystallizes in the monoclinic space group P2_1_/c with a = 9.699(1), b = 8.500(1), c = 18.044(2) Å, β = 101.290(7)° and Z = 4. The crystal structure was solved to final values R1 = 0.037 (for 2456 observed rfls. {I > 2σ[I]}) and wR2 = 0.102 (all data), see Additional file 1: Original X-ray analysis data by CIF format.

**Table 1:** **Crystal data and structure refinement details for 5-Methoxy-5,6-diphenyl-4,5-dihydro-2*H*-1,2,4-triazine-3-thione**

	
Empirical formula	C_16_H_15_N_3_OS
Formula weight	297.37
Temperature	193(2) K
Wavelength	0.71069 Å
Crystal system, space group	monoclinic, P2_1_/c
Unit cell dimensions	a = 9.699(1) Å
	b = 8.500(1) Å β = 101.290(7)°
	c = 18.044(2) Å
Volume	1458.8(3) Å^3^
Z, Calculated density	4, 1.354 Mg/m^3^
Absorption coefficient (MoKα )	0.224 mm^-1^
F(000)	624
Crystal size	0.6 × 0.5 × 0.5 mm
θ range for data collection	2.14 to 25.66 deg.
Limiting indices	−11 < =h < =11, -10 < =k < =10, -21 < =l < =21
Reflections collected / unique	2618 / 2456 [R(int) = 0.0396]
Completeness to theta = 28.04	95.0%
Absorption correction	Semi-empirical from equivalents
Refinement method	Full-matrix least-squares on F^2^
Data / restraints / parameters	2618 / 0 / 251
Goodness-of-fit on F^2^	1.062
Final R indices [for 2456 rfls with I > 2σ(I)]	R1 = 0.0371, wR2 = 0.1008
R indices (all data)	R1 = 0.0391, wR2 = 0.1024
Largest diff. peak and hole	0.322 and −0.305 e. Å^-3^
(Δ/δ)_max_	0.001

**Table 2:** **Atomic coordinates (Å × 10**^**4**^**) and equivalent isotropic displacement parameters (Å**^**2 **^**× 10**^**3**^**) for 5-Methoxy-5,6-diphenyl-4,5-dihydro-2*H*-1,2,4-triazine-3-thione**

	**X**	**Y**	**Z**	**U(eq)**
C(1)	−696(15)	3792(16)	7896(9)	22 (3)
C(2)	−1824(14)	2391(16)	8841(8)	21 (3)
C(4)	447(17)	1973(2)	9603(12)	35 (4)
C(5)	−3268(14)	1621(17)	8654(9)	22 (3)
C(6)	−3661(16)	440(18)	9104(10)	28(4)
C(7)	−5008(17)	−187(2)	8934(11)	34 (4)
C(8)	−5965(17)	366(2)	8323(11)	34 (4)
C(9)	−5577(18)	1524(2)	7869(11)	35 (4)
C(10)	−4230(17)	2147(19)	8036(10)	29 (4)
C(11)	−1928(14)	4053(17)	9152(9)	21 (3)
C(12)	−2516(14)	4336(18)	9839(9)	23 (3)
C(13)	−2303(16)	3307(2)	10454(9)	28 (4)
C(14)	−2771(18)	3707(2)	11109(10)	32 (4)
C(15)	−3463(17)	5101(2)	11161(10)	34 (4)
C(16)	−3700(17)	6122(2)	10550(11)	32 (4)
C(17)	−3235(15)	5741(19)	9895(10)	27 (3)
N(1)	−1237(13)	2508(15)	8152(8)	24 (3)
N(2)	−907(14)	5145(15)	8234(8)	27 (3)
N(3)	−1504(13)	5298(14)	8857(8)	25 (3)
S(1)	161(4)	3790(4)	7165(2)	29 (17)
O(1)	−959(10)	1409(12)	9364(6)	24 (3)

U(eq) is defined as one third of the trace of the orthogonalized Uij tensor.

**Figure 3:**
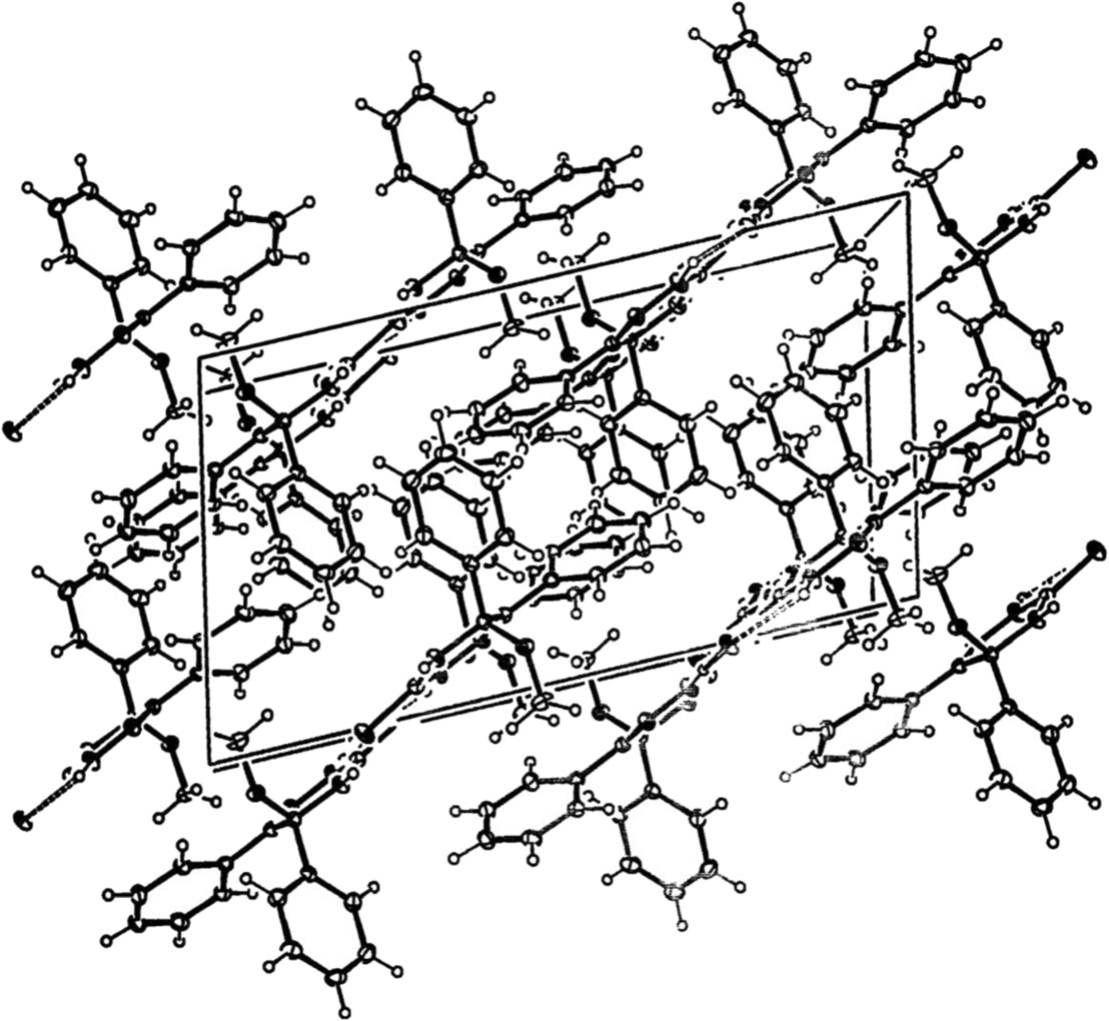
**Unit cell plot of 5-Methoxy-5,6-diphenyl-4,5-dihydro-2*H*-1,2,4-triazine-3-thione showing hydrogen bonding and π-π stacking interactions between these molecules.**

The dihedral angle between the planes of the triazine and two phenyl groups are 32.91 Å and 47.58 Å. The dihedral angle between the methoxyl group and the triazine is 61.69 Å. As shown in Figure 3, intermolecular hydrogen bonds are formed with NH groups acting as donors and S atoms as acceptors, N-H^…^S. The corresponding distances and angles for the hydrogen bonds are given in Table 3. There are both face-to-face and edge to face π-π stacking interactions between aromatic rings [8], as shown in Figure 3. The face-to-face and edge to face interplanar distances are 3.325 Å, appreciably shorter than the normal *π- π* stacking, and 3.704 Å, respectively.

**Table 3:** Hydrogen bond distances and angels (in Å and °, respectively) of 5-Methoxy-5,6-diphenyl-4,5-dihydro-2H-1,2,4-triazine-3-thione

**D-H**	**d(D-H)**	**d(H…A)**	**d(D…A)**	**DHA**	**A**
N(2)-H(2 N)	0.924	2.398	3.293	163.82	S(1) [−x,1/2 + y,1.5-z]

**Figure 4:**
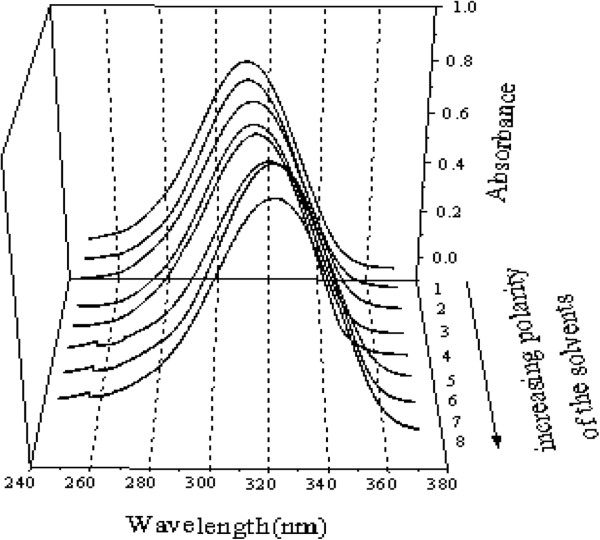
**The electronic absorption spectra of a 3.8 × 10**^**-5**^ **mol liter**^**-1 **^**5-Methoxy-5,6-diphenyl-4,5-dihydro-2*H*-1,2,4-triazine-3-thione in organic solvents:(1)C**_**6**_**H**_**12 **_**(2)CCl**_**4 **_**(3)CHCl**_**3 **_**(4)EtOH (5)MeOH (6)DMF (7)CH**_**3**_**CN (8)DMSO.**

The electronic absorption spectra of 5-Methoxy-5,6-diphenyl-4,5-dihydro-2H-1,2,4-triazine-3-thione was studied in organic solvents of different polarities, viz. Hexane, CCl_4_, CHCl_3_, C_2_H_5_OH, CH_3_OH, DMF, CH_3_CN, DMSO (Figure 4). This was done with the intention to investigate the solvatochromic behavior of this compound. While moving from the non-polar solvent to the polar one, the main intense band at 310 nm, which is due to the (π–π*) transition, is red shifted by 13 nm. Therefore, the absorption band of the title compound corresponding to the π–π* transition, shifts to longer wavelengths with an increase of dielectric constant of the solvents. This suggests stabilization of the electronic excited state relative to the ground state. Thus, the title compound showed positive solvatochromic behavior [9,10].

## Conclusions

In summary, a single crystal of 5-Methoxy-5,6-diphenyl-4,5-dihydro-2H-1,2,4-triazine-3-thione was prepared. The synthesized compound was characterized by X-ray analysis.

The packing of the resulting crystal clearly revealed that a one-dimensional network is formed owing to the presence of intermolecular weak hydrogen-bonding and S · · · H interactions between the parallel molecules. Also, there are face-to-face π-π stacking interactions between aromatic rings of the neighbor chains in this compound.

Further studies have been conducted for recording the electronic absorption spectra of this molecule in organic solvents of different polarities. The observation of the spectroscopic behavior in the presence of organic solvents indicates that this compound has a positive solvatochromic.

### Experimental

**Scheme 1:**
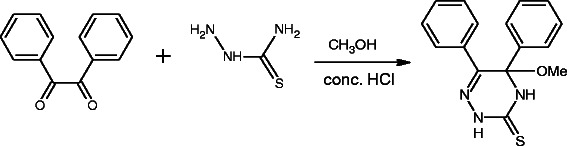
The synthesis procedure of the title compound.

The title compound was prepared by reaction of a solution of thiosemicarbazide (15 mmol) in methanol with a solution of HCl (30 cm^3^, 2 M), 1 cm^3^ of concentrated HCl and a solution of benzyl *(*15 mmol*)* in methanol at room temperature to form 5-Methoxy-5,6-diphenyl-4,5-dihydro-2*H*-1,2,4-triazine-3-thione [6]. M. P. = 232-233°C, Scheme 1, The branched tube method was used for the preparation of suitable single crystals [4]. The title compound (0.2 g) was placed in one arm of a branched tube, acetonitrile was carefully added to fill both arms, the tube sealed and the compound containing arm immersed in a bath at 60°C, while the other one was at ambient temperature. After 7 days, the suitable crystals for X-ray analysis of 5-Methoxy-5,6-diphenyl-4,5-dihydro-2*H*-1,2,4-triazine-3-thione were deposited in the cooler arm which was filtered off, washed with ether, and air dried.

## Competing interests

The authors declare that they have no competing interests.

## Authors’ contributions

BR designed and supervised the project. MF synthesized and characterized the compound. Both the authors read and approved the final manuscript.

## Supplementary Material

Additional file 1 **Complete bond lengths and angles, co-ordinates and displacement parameters have been deposited at Cambridge Crystallography Data Center.** Supplementary data are available from the CCDC, 12 Union Road, Cambridge CB2 1EZ, UK on request, quoting the deposition number 269111 for 5-Methoxy-5,6-diphenyl-4,5-dihydro-2*H*-1,2,4-triazine-3-thione. Click here for file
